# Anti-Inflammatory Properties of Oxygenated Isocoumarins and Xanthone from Thai Mangrove-Associated Endophytic Fungus *Setosphaeria rostrata*

**DOI:** 10.3390/molecules29030603

**Published:** 2024-01-26

**Authors:** Kedkarn Koopklang, Siwattra Choodej, Sujitra Hantanong, Ratchadaree Intayot, Siriporn Jungsuttiwong, Yuwadee Insumran, Nattaya Ngamrojanavanich, Khanitha Pudhom

**Affiliations:** 1 Program in Biotechnology, Faculty of Science, Chulalongkorn University, Bangkok 10330, Thailandsujittra_wu@hotmail.com (S.H.); 2 Department of Chemistry, Faculty of Science, King Mongkut’s University of Technology, Thonburi 10140, Thailand; 3 Department of Chemistry and Center of Excellence for Innovation in Chemistry, Faculty of Science, Ubon Ratchathani University, Ubon Ratchathani 34190, Thailand; 4 Department of Biology, Faculty of Science and Technology, Rajabath Maha Sarakham University, Maha Sarakham 44000, Thailand; 5 Department of Chemistry, Faculty of Science, Chulalongkorn University, Bangkok 10330, Thailand

**Keywords:** *Setosphaeria rostrata*, oxygenated isocoumarin, xanthone, endophytic fungus, anti-inflammation

## Abstract

Chronic inflammation plays a crucial role in the development and progression of numerous chronic diseases. To search for anti-inflammatory metabolites from endophytic fungi isolated from plants growing in Thai mangrove areas, a chemical investigation of those fungi was performed. Five new oxygenated isocoumarins, setosphamarins A–E (**1**–**5**) were isolated from the EtOAc extract of an endophytic fungus *Setosphaeria rostrata*, along with four known isocoumarins and one xanthone. Their structures were determined by extensive spectroscopic analysis. The absolute configurations of the undescribed compounds were established by comparative analysis between experimental and calculated circular dichroism (ECD) spectroscopy. All the compounds were evaluated for their anti-inflammatory activity by monitoring nitric oxide inhibition in lipopolysaccharide-induced macrophage J774A.1 cells. Only a xanthone, ravenelin (**9**), showed potent activity, with an IC_50_ value of 6.27 μM, and detailed mechanistic study showed that it suppressed iNOS and COX-2 expression.

## 1. Introduction

Chronic inflammation, a long-term response of a body to inflammatory stimuli, is recognized as a pathogenic core of various human disorders including cancer, arthritis, cardiovascular, asthma, and neurodegenerative diseases [1]. The highly reactive inflammatory factor, nitric oxide (NO), endogenously generated from oxidation of l-arginine catalyzed by nitric oxide synthase (NOS), is a crucial regulator in this complex process [2]. Indeed, there are three NOS forms, neuronal NOS (nNOS), inducible NOS (iNOS), and endothelial NOS (eNOS); however, only iNOS plays a pivotal role in immunity and inflammation process [3]. Moreover, evidence indicates that NO produced by iNOS plays a significant role in the activation of cyclooxygenase (COX) and inflammatory response, particularly neural inflammation [4]. In addition, there is a key relationship between COX and iNOS enzyme in pathologic conditions. During inflammation, iNOS increases the amount of cyclooxygenase-2 and, as a result, leads to increased effects of cytotoxic [3]. Consequently, inhibition of iNOS and COX-2 represents an attractive therapeutic development in the treatment of a diverse array of inflammatory conditions.

It has been widely recognized that endophytic fungi themselves are a potential source of bioactive metabolites, particularly those associated with an extreme environment, including mangrove forest [5]. The plants in those areas have special adaptations to survive in conditions of high salinity, extreme tides, strong winds, high temperatures, anaerobic soils, as well as a number of other environmental factors [6]. It is reasonable to expect mangrove plants to be habitats to a great variety of highly unique microbes including fungi [7]. For example, vermelhotin isolated from a marine-derived fungus *Nodulisporium* sp. was a potent anti-inflammatory agent suppressing the expression of iNOS, a key mediator of inflammation [8,9]. In our continuing effort to search for bioactive and new metabolites from plant-derived fungi, the chemical investigation of this exclusive fungal group collected from Thai mangrove forests was performed. A total of 13 fungal strains were isolated from *Ipomoea pes-caprae*, and an EtOAc crude extract obtained from the fungus SH8-8, further identified as *Setosphaeria rostrata* provided the interesting proton NMR profile, attracting our interest. Subsequent chemical investigation of the fungus led to the isolation and identification of five new isocoumarins (**1**–**5**), along with four known compounds (**6**–**9**). Herein, details of the isolation, structure elucidation and bioactivity of these compounds are reported.

## 2. Results and Discussion

**Figure 1:**
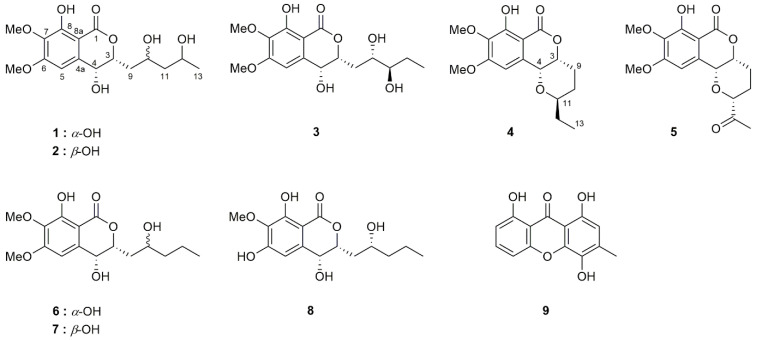
Structures of isolated compounds.

The SDB cultured broth extract of *S. rostrata* SH8-8 was subjected to various chromatographic techniques to afford a total of nine aromatic polyketides, including five new (**1**–**5**) and three known (**6**–**8**) isocoumarins and one known xanthone (**9**) (Figure 1). (3*R*,4*R*)-4,8-dihydroxy-3-((*R*)-2-hydroxypentyl)-6,7-dimethoxyisochroman-1-one (**6**) was obtained as a major metabolite (0.4 g, 7.8%) from 5.14 g of a crude broth extract of the SDB medium (10 L). The structures of the known compounds were confirmed by comparing their physicochemical and NMR spectroscopic data with those previously reported [10,11,12].

**Figure 2:**
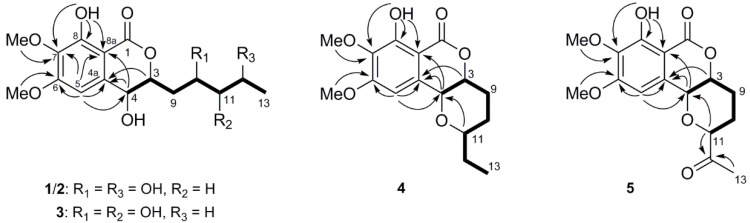
^1^H-^1^H COSY and key HMBC correlations of **1**–**5**.

**Figure 3:**
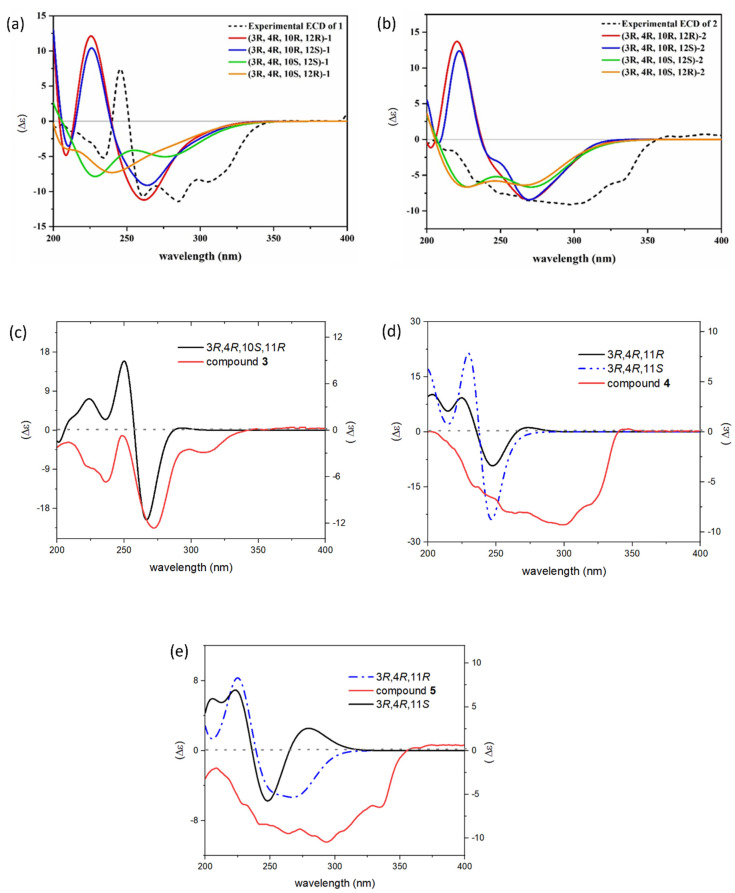
Experimental and calculated ECD spectra of compounds **1** (**a**), **2** (**b**), **3** (**c**), **4** (**d**) and **5** (**e**).

Setosphamarin A (**1**), obtained as a colorless oil, was established to have a molecular formula C_16_H_22_O_8_ by a protonated molecule at *m*/*z* 343.1361 [M + H]^+^ (calcd. for C_16_H_23_O_8_, 343.1393) in the HRESIMS spectrum, implying six degrees of unsaturation. The IR spectrum displayed hydroxyl and ester carbonyl absorption bands at 3409 and 1672 cm^−1^, respectively. The ^1^H NMR data of **1** (Table 1) displayed a chelated hydroxyl proton at δ_H_ 11.23 and a singlet aromatic proton at δ_H_ 6.57, which indicated the presence of a pentasubstituted phenolic ring. Combined analysis of 1D NMR and HSQC data revealed the presence of one primary methyl, two methoxyls, two methylenes, four oxymethines, six aromatic carbons (one unsubstituted), and one ester carbonyl carbon. Indeed, the NMR data, revealing that **1** contains an 8-hydroxyisochroman-1-one nucleus, closely resembled those of the main isocoumarin **6** [12], except a methylene in alkyl chain in **6** replaced by an oxymethine in **1**. Detailed inspection of the ^1^H-^1^H COSY data confirmed the presence of one isolated spin-system of CH-4/CH-3/CH_2_-9/CH-10/CH_2_-11/CH-12/CH_3_-13, indicating the replacement of the C-12 methylene in **6** by the oxymethine in **1**. The planar structure was further confirmed by the HMBC correlations of H-4 to C-4a and C-8a, H-5 to C-4, C-4a, C-6, C-7 and C-8a, 8-OH to C-7, C-8 and C-8a, 6-OMe to C-6, and 7-OMe to C-7 (Figure 2). The coupling constant *J*_3,4_ of 2.0 Hz indicated a *cis* relationship between H-3 and H-4. Moreover, observed NOESY correlation of H-3/H-4 indicated they were in the same orientation (Figure S4). To assign the absolute configuration of the compound, the electronic circular dichroism (ECD) calculation was performed using time-dependent density functional theory (TD-TDF). According to the results, the experimental ECD spectrum of compound **1** displayed similar curve shape and Cotton effects to those of the calculated ECD spectrum of the 3*R*, 4*R*, 10*R* configuration shown in Figure 3. However, the absolute configuration of the C-12 could not be determined, as the calculated ECD curve was not affected by its configuration. This might be because of the high flexibility of the chain [10].

Setosphamarin B (**2**), obtained as a colorless oil, had the same molecular formula C_16_H_22_O_8_ as compound **1** obtained by the HRESIMS data. The ^1^H and ^13^C NMR data of **2** closely resembled those of **1** (Table 1), with obvious differences for the ^13^C NMR signals for C-10, C-12 and C-13. The chemical shift of C-10 and C-12 was deshielded by 2.2 and 2.5 ppm, respectively, while that of C-13 was shielded by 5.7 ppm. In addition, the identical planar structure to **1** was established via the 2D NMR data, confirmed by ^1^H-^1^H COSY correlations of H-4/H-3/H_2_-9/H-10/H_2_-11/H-12/H_3_-13 and HMBC correlations and by HMBC correlations of H-4 to C-4a and C-8a, H-5 to C-4, C-4a, C-6, C-7 and C-8a, 8-OH to C-7, C-8 and C-8a, 6-OMe to C-6, and 7-OMe to C-7. The same *cis* configuration of H-3 and H-4 was assigned from the coupling constant *J*_3,4_ of 2.8 Hz. This suggests that **2** is a stereoisomer of **1**, with the difference in configuration at C-10 and/or C-12. Similarly, the absolute configuration of **2** was established based on comparison of its ECD curve with the calculated curve (Figure 3). Thereby the absolute structure of **2** was defined as 3*R*, 4*R*, 10*S*, and the configuration of the C-12 could not be determined with the same reason for **1**.

Setosphamarin C (**3**) was isolated as a colorless oil and was shown to have the same molecular formula as C_16_H_22_O_8_ as **1** and **2**, according to its HRESIMS data. Detailed inspection of the NMR data showed the structure of **3** resembled those of **1** and **2** (Table 1). The obvious distinction in the ^1^H NMR spectra was the splitting pattern of the C-13 methyl peak, due to the doublet in **1** and **2**, and the triplet in **3**. These implied that compound **3** was a C-11 hydroxy derivative of compound **6**, which was established and confirmed by a sequential spin-system of H-4/H-3/H_2_-9/H-10/H-11/H_2_-12/H_3_-13 in the ^1^H-^1^H COSY spectrum (Figure 2). Similarly, the *cis* configurations between H-3 and H-4 were assigned by the small coupling constant (*J*_3,4_ = 1.6 Hz). The absolute configuration of **3** was also determined by comparing experimental and calculated ECD spectra (Figure 3). The results reveal that the configuration of the C-3, C-4, C-10 and C-11 were *R*, *R*, *S*, and *R*, respectively.

Setosphamarin D (**4**) was isolated as a white powder. The molecular formula was established as C_16_H_20_O_6_ based on its HRESIMS analysis, indicating seven degrees of unsaturation. The similarity of the ^1^H and ^13^C NMR spectra (Table 1 and Table 2) of **1**–**3** and **4** suggests that **4** has the same 8-hydroxy-6,7-dimethoxyisochroman-1-one skeleton. The most significant difference was the replacement of a C-10 oxygenated methine of **1**–**3** by a methylene (δ_H_ 40.5) in **4**. In addition, the HMBC correlation of H-11with C-4, combined with its molecular formula C_16_H_20_O_6_, was suggestive of a ring closure to a tetrahydropyran ring (Figure 2). Additionally, the *cis* configuration between H-3 and H-4 was assigned based on a small coupling constant (*J* = 2.0 Hz). In addition, the NOESY spectrum of **4** showed cross-peaks from H-3 to H-4, but those from H-3 and H-4 to H-11 were not observed, indicating that H-3 and H-4 are α-oriented, and that H-11 is β-oriented (Figure S22). The absolute configuration of **4** was established as 3*R*, 4*R*, 11*R* by comparing experimental and calculated ECD spectra (Figure 3).

Setosphamarin E (**5**) was isolated as a colorless oil, with the molecular formula C_16_H_18_O_7_ (eight degrees of unsaturation) deduced from the HRESIMS at *m*/*z* 323.1160 [M + H]^+^ (calcd for C_16_H_19_O_7_, *m/z* 323.1131). The NMR spectra of **5** (Table 2) were similar to those of **4**. The major difference was the absence of a methylene (C-12) in **4**, which was replaced by a carbonyl ketone carbon (δ_C_ 206.2) in **5**. Additionally, significant differences of the signals for C-9, C-11 and C-13 were observed. This assignment was confirmed by its HMBC cross-peaks with H_3_-13 and H-11, together with the correlations in the ^1^H-^1^H COSY spectrum of H-4/H-3/H_2_-9/H_2_-10/H-11 (Figure 2). The *cis* configuration between H-3 and H-4 was determined with the small coupling constant (*J*_3,4_ 2.0 Hz). The absolute configuration of **5** was determined as 3*R*, 4*R*, 11*R* by comparing experimental and calculated ECD spectra (Figure 3).

**Figure 4:**
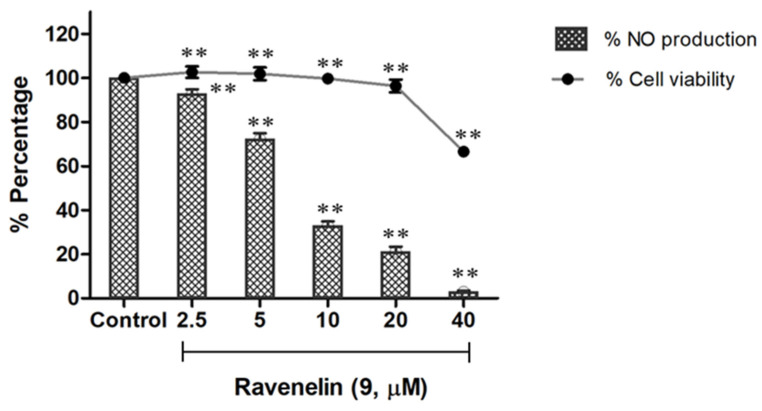
Effect of ravenelin (**9**) on NO production of J744.A1 cells and cell viability. ** *p* < 0.01 versus control.

NO is the major pro-inflammatory cytokine playing a crucial role in the inflammatory response [13,14]. Moreover, NO is also a key mediator for numerous neurodegenerative diseases, including Alzheimer’s disease (AD), Parkinson’s disease (PD), and ischemic brain injury (stroke) [15]. This has prompted scientists to pay attention to search for and develop effective molecules for NO inhibition. All of the compounds were thus subjected to NO inhibitory assay in lipopolysaccharide (LPS)-activated macrophage J774A.1 cells to evaluate their anti-inflammatory activity. Additionally, the cell viability was determined by the MTT assay. Treatment with ravenelin (**9**), a xanthone, displayed remarkable activity from a dose of 5 μM, providing an IC_50_ value of 6.27 μM (Figure 4), while indomethacin was used as a positive control with IC_50_ value of 41.01 μM (Table 3). Furthermore, the compound did not show any detectable toxicity to the cells at concentrations ranging from 2 to 20 μM with cell viability >95%; however, cytotoxicity was observed in doses of up to 40 μM (Figure 4). Among isocoumarin derivatives, compounds **1**–**3** showed comparable activity to indomethacin with IC_50_ values ranging from 23.17 to 35.79 μM, whereas isocoumarins **4**–**8** did not show any significant activity at a screening dose of 50 μM.

**Figure 5:**
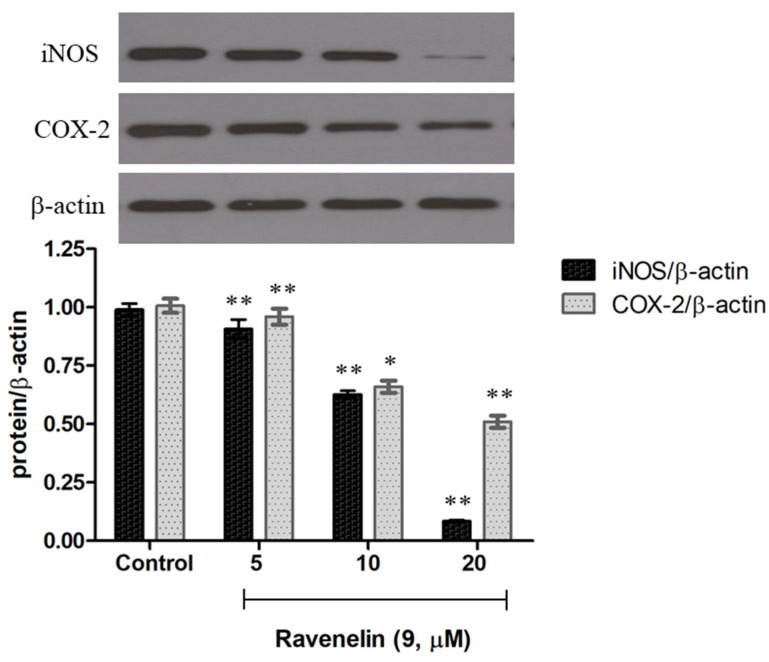
Effect of ravenelin (**9**) on iNOS and COX-2 protein expression. * *p* < 0.05; ** *p* < 0.01 versus control.

Further, Western blotting was performed to examine whether **9** can reduce iNOS and COX-2 expression, the protein expression of inflammation-associated molecules triggered by LPS [16]. J774A.1 cells were treated with LPS with or without the indicated concentrations of **9** to observe the effect on iNOS and COX-2 expression. Notably, upon treatment with only LPS, the protein expression level of iNOS and COX-2 increased markedly (control), while pretreatment of the cells with different concentrations of **9** led to a decrease in both iNOS and COX-2 expression in a dose-dependent manner (Figure 5). These data suggest that ravenelin **9** possessed a potent anti-inflammatory activity, which is mediated mainly by the suppression of iNOS and COX-2 expression.

## 3. Material and Methods

### 3.1. General Experimental Procedures

The optical rotation was measured on a JASCO P-1010 (JASCO, Easton, MD, USA). UV data were recorded on a CARY 50 Probe UV-visible spectrophotometer (Agilent Technologies, Santa Clara, CA, USA). The IR spectra were recorded using a PerkinElmer FTS FT-IR spectrophotometer (PerkinElmer, Shelton, CT, USA). Electronic circular dichroism (ECD) spectra were recorded on a JASCO J-815 CD spectropolarimeter (JASCO, Easton, MD, USA). The NMR spectra were recorded on a Bruker AV400 (400 MHz for ^1^H-NMR, 100 MHz for ^13^C-NMR; Bruker, Billerica, MA, USA) using tetramethylsilane (TMS) as an internal standard. Silica gel G60 (60–200 mm; SiliCycle Inc., Quebec, QC, Canada), and Sephadex LH-20 (18–111 µm, GE Healthcare, Chicago, IL, USA) were used for column chromatography (CC). HRESIMS spectra were obtained with a Bruker micrOTOF-Q II (Bruker, Billerica, MA, USA). TLC normal and TLC reverse phase analysis were performed on Silicycle’s aluminum sheet coated with silica gel F-254 (SiliCycle Inc., Quebec, QC, Canada) and on Merck’s aluminum sheets coated with silica gel 60 RP-18 F_245_s (Merck, Rahway, NJ, USA), respectively.

### 3.2. Biological Materials

The fungal strain SH8-8 was isolated from *Ipomoea pes-caprae* leaves, which were collected from a mangrove forest in Pran Buri District, Prachuap Kiri Khan Province, Thailand. The fungus was stored on PDA slants at 4 °C and deposited at the Department of Chemistry, Faculty of Science, Chulalongkorn University. The fungus SH8-8 was identified as *Setosphaeria rostrata* based on its morphology and the rDNA internal transcribed spacers sequence (GenBank accession OK047731).

### 3.3. Fermentation, Extraction and Isolation

The fungal strain SH8-8 was grown on PDA plates at room temperature for 5 days, then inoculated into a 1000 mL Erlenmeyer flask containing 250 mL of SDB medium (SDB 30 g, distilled water 1 L). The flasks were statically incubated at room temperature under a normal day-night cycle. After 21-day incubation, the culture was filtered, and the broth was then extracted with EtOAc to afford 5.14 g of the extract. The EtOAc crude extract was fractionated by silica gel (SiO_2_) column chromatography (CC), eluted by gradient mixtures of EtOAc–hexane (from 1:9 to 1:0 *v*/*v*) followed by MeOH–EtOAc (from 2:8 to 1:1 *v*/*v*) to give 15 fractions (F1-F15). Fraction F5 (203 mg) was subjected SiO_2_ flash CC, eluted with acetone-CH_2_Cl_2_ mixtures (from 2:98 to 4:96 *v*/*v*), then subfraction F5S1 (41 mg) was purified by SiO_2_ CC (CH_2_Cl_2_–hexane, 1:1 *v*/*v*) to obtain compound **9** (5.4 mg). Fraction F6 (1.68 g) was further separated by SiO_2_ CC eluted with EtOAc–hexane (2:3 *v*/*v*) to yield compound **6** (329.7 mg), while fraction F7 (50.4 mg) was further purified over a SiO_2_ column with EtOAc–CH_2_Cl_2_ mixtures (from 2:98 to 6:94 *v*/*v*) to give compounds **6** (70.3 mg) and five subfractions, of which F7S1 (28.0 mg) gave compound **8** (9.3 mg) with gradient mixtures of EtOAc–CH_2_Cl_2_ (from 2:98 to 4:96 *v*/*v*). Fraction F12 (227.5 mg) was subjected to SiO_2_ CC, eluted with gradient mixtures of (EtOAc–CH_2_Cl_2_, from 3:7 to 6:4 *v*/*v*) to give three fractions (F12S1-F12S3). The fraction F12S2 (92.9 mg) was further separated by SiO_2_ CC with acetone–CH_2_Cl_2_ mixtures (from 1:9 to 2:8 *v*/*v*) followed by Sephadex LH-20 CC with MeOH to afford compound **4** (4.7 mg). Subfraction F12S3 (12.7 mg) was applied to ODS CC eluted with gradients of MeOH–H_2_O (from 1:0 to 0:1 *v*/*v*) to furnish compounds **4** (4.8 mg) and **5** (1.3 mg). Fraction F13 (159.5 mg) was rechromatographed on a Sephadex LH-20 column eluted with MeOH to afford **3** (2.7 mg), and separation of subfraction F13P2 (12.9 mg) with a 2:98 mixture of MeOH–CH_2_Cl_2_ yielded compound **7** (4.2 mg). Finally, fraction F14 (646.9 mg) was further separated on a Sephadex LH-20 column with MeOH to give four subfractions, F14P1-F14P4. Subfraction F14P1 (222.3 mg) was further subjected to SiO_2_ CC (MeOH−CH_2_Cl_2_, from 1:99 to 4:96 *v*/*v*), followed by ODS CC with gradient mixtures of MeOH−H_2_O (from 1:1 to 0:1 *v*/*v*) to give compounds **1** (4.2 mg) and **2** (7.1 mg).

*Setosphamarin A* (**1**), colorless oil; ${\left[\alpha \right]}_{D}^{20}$ − 94 (*c* 0.1, MeOH); UV (MeOH) *λ*_max_ (log *ε*) 232 (3.74), 274 (1.18), 307 (0.46) nm; ECD (acetonitrile) *λ*_max_ (*mdeg*) 245 (+7.4), 285 (−11.4), 340 (−0.6) nm; IR *ν*_max_ 3409, 2930, 2866, 1672, 1434, 1284, 1150, 960 cm^−1^; ^1^H-NMR (400 MHz, CDCl_3_) and ^13^C-NMR (100 MHz, CDCl_3_) (Table 1); HRESIMS *m/z* 343.1361 [M + H]^+^ (calcd. for C_16_H_23_O_8_, 343.1393).
*Setosphamarin B* (**2**), colorless oil; ${\left[\alpha \right]}_{D}^{20}$ + 56 (*c* 0.1, MeOH); UV (MeOH) *λ*_max_ (log *ε*) 231 (4.10), 274 (1.22), 306 (0.51) nm; ECD (acetonitrile) *λ*_max_ (*mdeg*) 234 (−5.9), 297 (−9.1), 388 (+0.7) nm; IR *ν*_max_ 3406, 2926, 2856, 1672, 1430, 1284, 1140, 956 cm^−1^; ^1^H-NMR (400 MHz, CDCl_3_) and ^13^C-NMR (100 MHz, CDCl_3_) (Table 1); HRESIMS *m/z* 343.1374 [M + H]^+^ (calcd. for C_16_H_23_O_8_, 343.1393).
*Setosphamarin C* (**3**), colorless oil; ${\left[\alpha \right]}_{D}^{20}$ + 118 (*c* 0.1, MeOH); UV (MeOH) *λ*_max_ (log *ε*) 231 (3.96), 273 (0.98), 307 (0.41) nm; ECD (acetonitrile) *λ*_max_ (*mdeg*) 245 (−2.1), 272 (−11.9), 328 (−0.6) nm; IR *ν*_max_ 3398, 2926, 2871, 1670, 1452, 1279, 1141, 949 cm^−1^; ^1^H-NMR (400 MHz, CDCl_3_) and ^13^C-NMR (100 MHz, CDCl_3_) (Table 1); HRESIMS *m/z* 343.1411 [M + H]^+^ (calcd. for C_16_H_23_O_8_, 343.1393).
*Setosphamarin D* (**4**), white powder; ${\left[\alpha \right]}_{D}^{20}$ − 11 (*c* 0.1, MeOH); UV (MeOH) *λ*_max_ (log *ε*) 232 (3.77), 274 (0.87), 307 (0.42) nm; ECD (acetonitrile) *λ*_max_ (*mdeg*) 234 (−5.4), 297 (−9.2), 346 (+0.2) nm; IR *ν*_max_ 3426, 2941, 2868, 1675, 1451, 1281, 1136, 956 cm^−1^; ^1^H-NMR (400 MHz, CDCl_3_) and ^13^C-NMR (100 MHz, CDCl_3_) (Table 2); HRESIMS *m/z* 309.1120 [M + H]^+^ (calcd. for C_16_H_21_O_6_, 309.1138).
*Setosphamarin E* (**5**), colorless oil; ${\left[\alpha \right]}_{D}^{20}$ + 44 (*c* 0.1, MeOH); UV (MeOH) *λ*_max_ (log *ε*) 232 (3.94), 274 (1.00), 307 (0.63) nm; ECD (acetonitrile) *λ*_max_ (*mdeg*) 234 (−5.4), 297 (−9.2), 346 (+0.2) nm; IR *ν*_max_ 3411, 2962, 2850, 1742, 1669, 1460, 1273, 1147, 968 cm^−1^; ^1^H-NMR (400 MHz, CDCl_3_) and ^13^C-NMR (100 MHz, CDCl_3_) (Table 2); HRESIMS *m/z* 323.1160 [M + H]^+^ (calcd. for C_16_H_19_O_7_, 323.1131).

### 3.4. ECD Calculation

Absolute configurations of compounds **1**–**5** were assigned by ECD calculation performed by time-dependent density functional theory (TD-DFT) calculations at the B3LYP functional with level of 6-311++G (d,p). DFT geometry optimization were run at the B3LYP/6-311++G (d,p) level of theory, including the integral equation formalism of the conductor-like polarizable continuum solvation model (C-PCM) for methanol. The rotational strengths were calculated for 90 excited states. Gaussian band shape with a 0.30 eV bandwidth was executed to simulate ECD spectra. Theoretical ECD curves were generated by the software SpecDis 1.71 (University of Wurzburg, Wurzburg, Germany). All calculations were performed using GAUSSIAN 16 Rev. C.01 program (Gaussian, Inc., Willford, CT, USA).

### 3.5. Anti-Inflammatory Assay

Anti-inflammatory assay of the compounds was determined by NO inhibition assay as described previously [17]. The positive control was indomethacin, a nonsteroidal anti-inflammatory drug. Mouse macrophage J774A.1 cells (ATCC^®^ TIB-67™, ATCC, Manassas, VA, USA) were seeded into each well of a 24-well plate at a density of 1 × 10^5^ cells/well and incubated at 37 °C and 5% CO_2_ for 24 h. The cells were treated with test compounds at various concentrations and vehicle (DMSO) for 2 h, then stimulated with LPS (1 μg/mL) for an additional 20 h. The cell supernatants (50 μL) of each well were collected and mixed with Griess reagent and the absorbance was measured at 540 nm with a microplate reader (PowerWave XS2 Bio-Tek Instrument, Agilent Technologies, Santa Clara, CA, USA). The amount of nitrite was calculated from a standard curve of known nitrite concentration.

### 3.6. Cytotoxicity Assay

To determine toxicity of the compounds on macrophage J774A.1, cell viability was assessed under the same experimental conditions in anti-inflammatory assay by 3-(4,5-dimethylthiazol-2-yl)-2,5-diphenyltetrazolium bromide (MTT) assay as described previously [17]. J774A.1 cells were treated with test compounds at various concentrations and vehicles (DMSO) at 37 °C and 5% CO_2_ for 20 h. MTT solution (final concentration 0.5 mg/mL) was added to each well and incubated for another 4 h. After removing the medium, DMSO (200 μL) was added to dissolve the formed formazan, and the absorbance was measured at 570 nm.

### 3.7. Western Blot

Cells treated as indicated in the text were washed with cold PBS and lysed with 1× cell lysis buffer (mammalian protein extraction buffer, GE Healthcare, USA), according to the manufacturer’s instructions. After 5 min incubation, cells were centrifuged at 14,000 rpm and at 4 °C for 5 min, then cell lysate was collected and stored at −80 °C until use. Equal amounts (30 μg) of total protein in each cell lysate were resolved by 10% SDS-PAGE and transferred to a PVDF membrane. To block nonspecific protein binding, the membrane was incubated with 3% skim milk in PBS containing 0.05% Tween-20 for 1 h at room temperature. The membrane was further incubated with the corresponding primary antibodies (Cell Signaling Technology, Danvers, MA, USA) in 3% skim milk in PBS containing 0.05% Tween-20 overnight at 4 °C, followed by incubation with horseradish peroxidase-conjugated secondary antibody for 1 h at room temperature. The signals were then detected using a chemiluminescence method.

### 3.8. Statistical Analysis

Experiments were independently performed in triplicate (*n* = 3) and data are presented as mean values ± standard deviation, analyzed using ANOVA and Dunnett’s multiple comparison test by GraphPad Prism 7.0 software. A *p* value of < 0.05 was considered to be statistically significant.

## 4. Conclusions

In summary, we have isolated five new isocoumarins (**1**–**5**), namely setosphamarins A–E, along with four known compounds (**6**–**9**) from the SDB broth extract of the mangrove-associated fungus *S. rostrata*. The structures of the new compounds were deduced by extensive analysis of their spectroscopic data, and the absolute configurations were defined by comparison between the experimental and calculated ECD curves. All of the compounds were assayed for their anti-inflammatory activity by monitoring their potency on NO production in LPS-activated macrophage cells. The results reveal that ravenelin (**9**), a xanthone, showed the most potent activity with IC_50_ values of 6.27 μM, compared with a positive control, indomethacin. Moreover, this study has demonstrated that its effect was mainly mediated by suppression of iNOS and COX-2 expression. Thus, the compound may have the potential to become a therapeutic drug for inflammation-related diseases.

## Figures and Tables

**Table 1:** ^1^H (δ in ppm, *J* in Hz) and ^13^C NMR data of compounds **1**–**3** in CDCl_3_.

No.	1		2		3	
	δ_H_	δ_C_	δ_H_	δ_C_	δ_H_	δ_C_
1		167.8		167.7		167.2
3	5.06, m	80.8	5.04, m	81.2	5.19, m	81.1
4	4.61, d (2.0)	75.0	4.54, d (2.8)	74.7	4.62, d (2.0)	75.2
4a		130.7		130.9		130.8
5	6.57, s	104.5	6.56, s	104.5	6.60, s	104.7
6		158.7		158.7		158.9
7		137.9		137.5		137.5
8		156.3		156.3		156.4
8a		102.3		102.0		102.1
9	2.20, m	39.6	2.18, m	39.5	2.28, m	36.1
	2.67, m		2.64, m		2.56, m	
10	4.35, m	78.4	4.39, m	75.9	3.98, m	82.0
11	1.68, m	44.9	1.71, m	44.6	3.53, m	74.8
	1.77, m		1.84, m			
12	3.99, m	67.2	3.99, m	65.0	1.48, m	26.1
					1.52, m	
13	1.19, d (7.6)	23.6	1.19, d (7.6)	29.3	1.01, t (8.0)	10.0
6-OMe	3.95, s	56.3	3.93, s	56.6	3.96, s	56.5
7-OMe	3.91, s	60.7	3.88, s	60.9	3.91, s	60.9
8-OH	11.23, s		11.23, s		11.23, s	

**Table 2:** ^1^H (δ in ppm, *J* in Hz) and ^13^C NMR data of compounds **4**–**5** in CDCl_3_.

No.	4		5	
	δ_H_	δ_C_	δ_H_	δ_C_
1		168.8		167.7
3	4.74, m	79.4	4.58, overlap	74.2
4	4.66, d (2.0)	66.9	4.58, d (2.8)	74.7
4a		136.4		130.8
5	6.56, s	102.8	6.59, s	104.6
6		158.8		158.8
7		137.0		137.5
8		156.1		156.3
8a		101.9		101.9
9	1.87, m	37.7	2.75, overlap	49.8
	2.17, m		2.98, dd (6.0, 17.6)	
10	1.53, m	40.5	2.18, m	39.2
			2.74, overlap	
11	3.89, overlap	67.8	5.05, dd (2.8, 5.2)	81.2
12	1.40, m	18.6		206.2
13	0.96, t (7.6)	13.9	2.14, s	30.6
6-OMe	3.94, s	56.3	3.94, s	56.3
7-OMe	3.89, s	60.7	3.89, s	60.7
8-OH	11.07, s		11.20, s	

**Table 3:** Nitric oxide inhibitory activity of **1**–**9**.

Compounds	IC_50_ (μM) ^a^
**1**	23.17 ± 1.19 **
**2**	35.79 ± 1.79 **
**3**	25.46 ± 1.41 *
**4**	>50
**5**	>50
**6**	>50
**7**	>50
**8**	>50
**9**	6.27 ± 0.87 **
Indomethacin	41.01 ± 1.87 **

* *p* < 0.05, ** *p* < 0.01 versus control. ^a^ The IC_50_ values are represented as the means ± SD (*n* = 3).

## Data Availability

The data presented in this study are available in the present article and the Supplementary Material.

## Supplementary Materials

The following supporting information can be downloaded at: https://www.mdpi.com/article/10.3390/molecules29030603/s1, Figures S1–S29: 1D and 2D NMR spectra of compounds **1**–**5**.
