# Supplementary material for: Anti-Inflammatory Properties of Oxygenated Isocoumarins and Xanthone from Thai Mangrove-Associated Endophytic Fungus Setosphaeria rostrata
Source: Molecules. 2024 Jan 26;29(3):603. doi: 10.3390/molecules29030603 (PMC10856793; doi:10.3390/molecules29030603)
Supplement: Supplementary file 1 [file molecules-29-00603-s001.zip › molecules-2760443-supplementary.pdf]

Supporting Information

Anti-inflammatory properties of oxygenated isocoumarins and xanthone from Thai mangrove-associated endophytic fungus *Setosphaeria rostrata*

Kedkarn Koopklang[1], Siwattra Choodej[2], Sujitra Hantanong[1], Ratchadaree Intayot[3], Siriporn Jungsuttiwong[3], Yuwadee Insumran[4], Nattaya Ngamrojanavanich[6] and Khanitha Pudhom[6,*]

[1] Program in Biotechnology, Faculty of Science, Chulalongkorn University, Bangkok 10330, Thailand

[2] Department of Chemistry, Faculty of Science, King Mongkut's University of Technology Thonburi, 10140, Thailand

[3] Department of Chemistry and Center of Excellence for Innovation in Chemistry, Faculty of Science, Ubon Ratchathani University, Ubon Ratchathani 34190, Thailand

[4] Department of Biology, Faculty of Science and Technology, Rajabath Maha Sarakham University, Maha Sarakham 44000, Thailand

[5] Department of Chemistry, Faculty of Science, Chulalongkorn University, Bangkok 10330, Thailand

---

Corresponding author.

E-mail addresses: Khanitha.P@chula.ac.th (K. Pudhom).

Table of Contents

**Fig. S1.**	^{1}H NMR spectrum (400 MHz, $CDCl_3$) of setosphamarin A (**1**)	8
**Fig. S2.**	^{13}C NMR spectrum (100 MHz, $CDCl_3$) of setosphamarin A (**1**)	8
**Fig. S3.**	COSY spectrum of setosphamarin A (**1**)	9
**Fig. S4.**	NOESY spectrum of setosphamarin A (**1**)	9
**Fig. S5.**	HSQC spectrum of setosphamarin A (**1**)	10
**Fig. S6.**	HMBC spectrum of setosphamarin A (**1**)	10
**Fig. S7.**	^{1}H NMR spectrum (400 MHz, $CDCl_3$) of setosphamarin B (**2**)	11
**Fig. S8.**	^{13}C NMR spectrum (100 MHz, $CDCl_3$) of setosphamarin B (**2**)	11
**Fig. S9.**	COSY spectrum of setosphamarin B (**2**)	12
**Fig. S10.**	NOESY spectrum of setosphamarin B (**2**)	12
**Fig. S11.**	HSQC spectrum of setosphamarin B (**2**)	13
**Fig. S12.**	HMBC spectrum of setosphamarin B (**2**)	13
**Fig. S13.**	^{1}H NMR spectrum (400 MHz, $CDCl_3$) of setosphamarin C (**3**)	14
**Fig. S14.**	^{13}C NMR spectrum (100 MHz, $CDCl_3$) of setosphamarin C (**3**)	14
**Fig. S15.**	COSY spectrum of setosphamarin C (**3**)	15
**Fig. S16.**	NOESY spectrum of setosphamarin C (**3**)	15
**Fig. S17.**	HSQC spectrum of setosphamarin C (**3**)	16
**Fig. S18.**	HMBC spectrum of setosphamarin C (**3**)	16
**Fig. S19.**	^{1}H NMR spectrum (500 MHz, $CDCl_3$) of setosphamarin D (**4**)	17
**Fig. S20.**	^{13}C NMR spectrum (125 MHz, $CDCl_3$) of setosphamarin D (**4**)	17
**Fig. S21.**	COSY spectrum of setosphamarin D (**4**)	18
**Fig. S22.**	NOESY spectrum of setosphamarin D (**4**)	18
**Fig. S23.**	HSQC spectrum of setosphamarin D (**4**)	19
**Fig. S24.**	HMBC spectrum of setosphamarin D (**4**)	19
**Fig. S25.**	^{1}H NMR spectrum (500 MHz, $CDCl_3$) of setosphamarin E (**5**)	20
**Fig. S26.**	^{13}C NMR spectrum (125 MHz, $CDCl_3$) of setosphamarin E (**5**)	20
**Fig. S27.**	COSY spectrum of setosphamarin E (**5**)	21
**Fig. S28.**	HSQC spectrum of setosphamarin E (**5**)	21
**Fig. S29.**	HMBC spectrum of setosphamarin E (**5**)	22

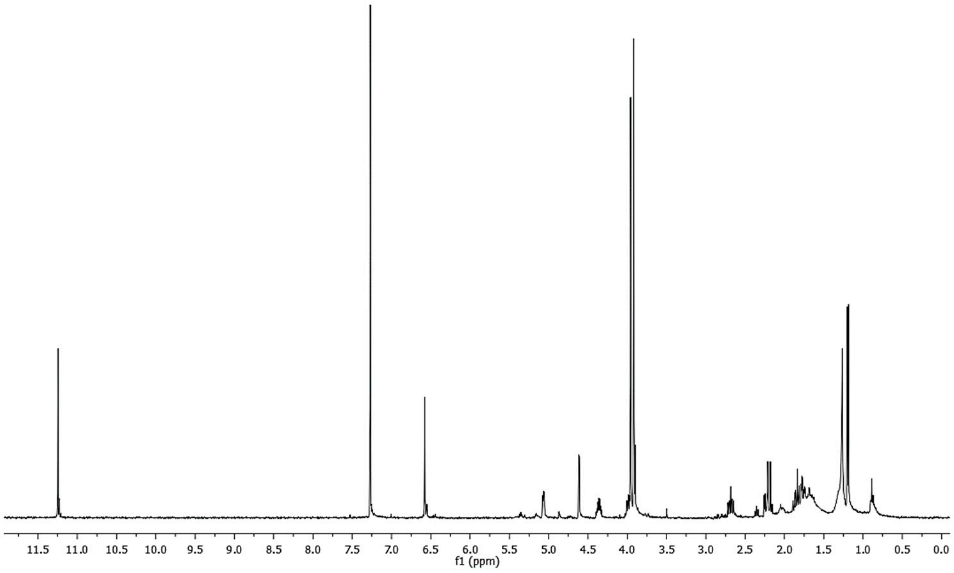

Fig. S1. ^{1}H NMR spectrum (400 MHz, $CDCl_3$) of setosphamarin A (**1**).

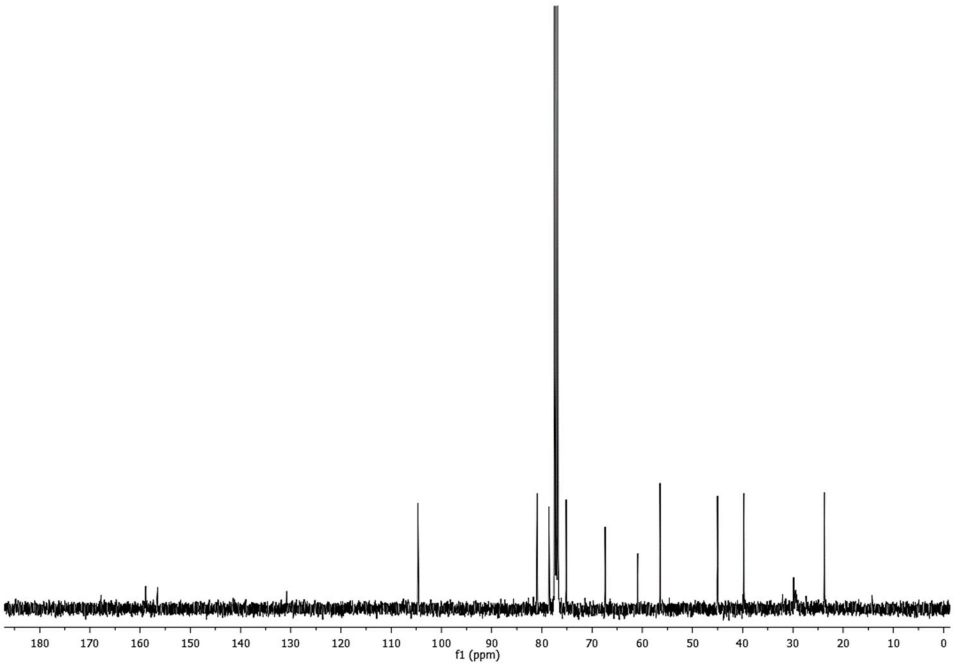

Fig. S2. ^{13}C NMR spectrum (100 MHz, $CDCl_3$) of setosphamarin A (**1**).

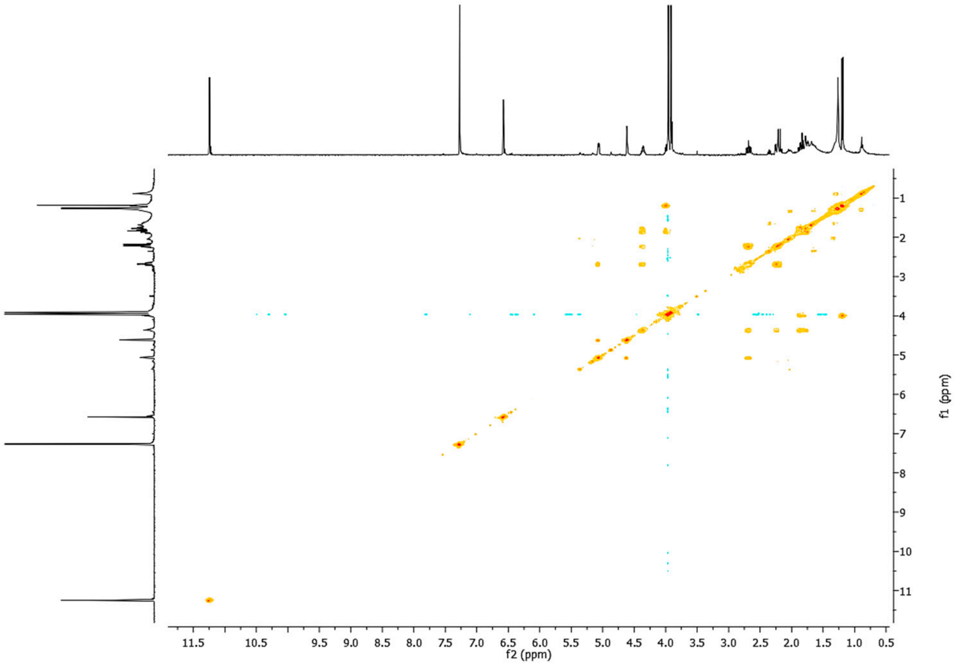

Fig. S3. COSY spectrum of setosphamarin A (**1**)

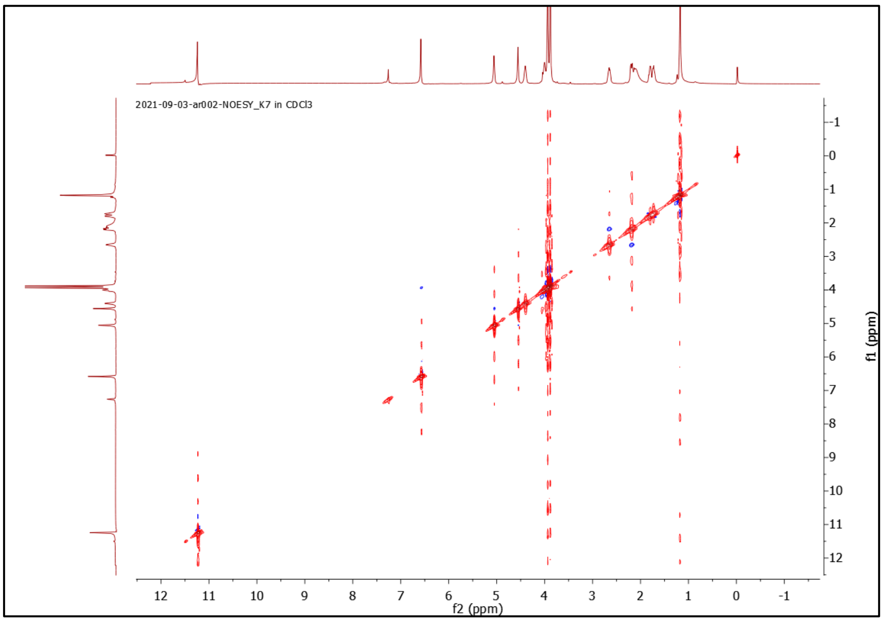

Fig. S4. NOESY spectrum of setosphamarin A (**1**)

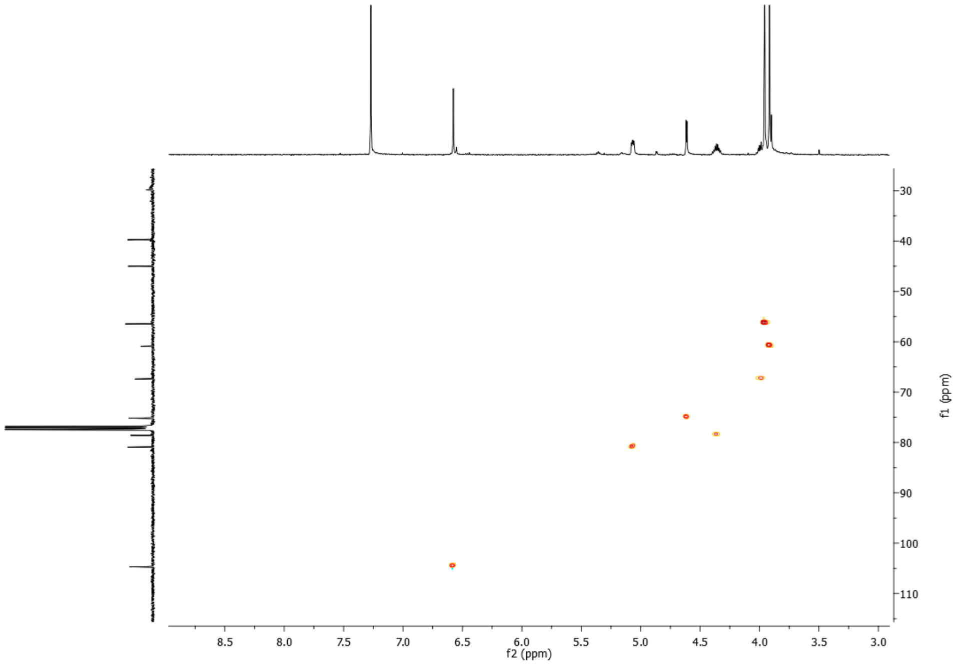

Fig. S5. HSQC spectrum of setosphamarin A (**1**)

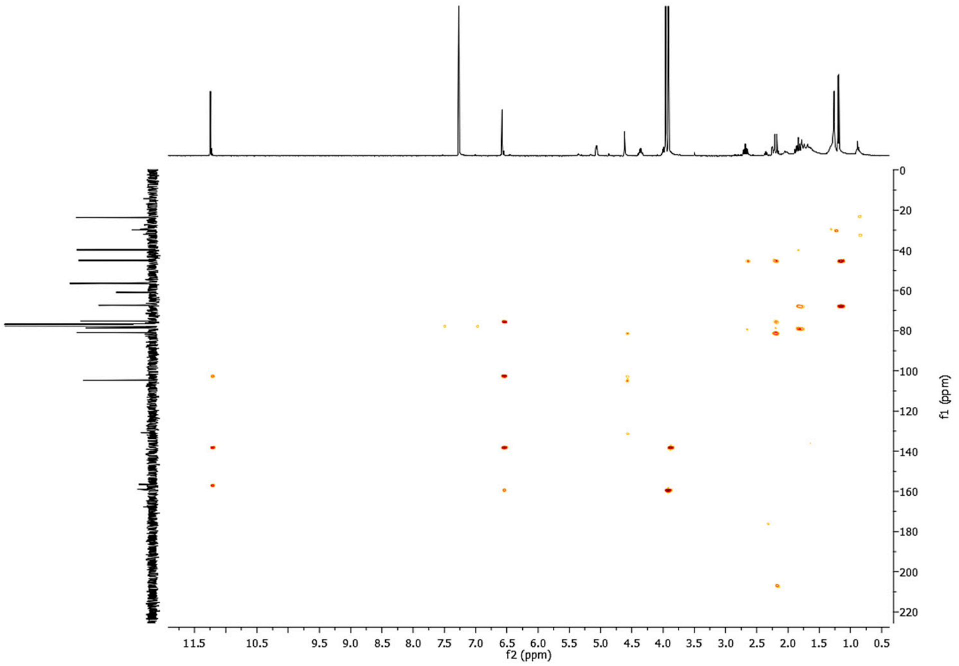

Fig. S6. HMBC spectrum of setosphamarin A (**1**)

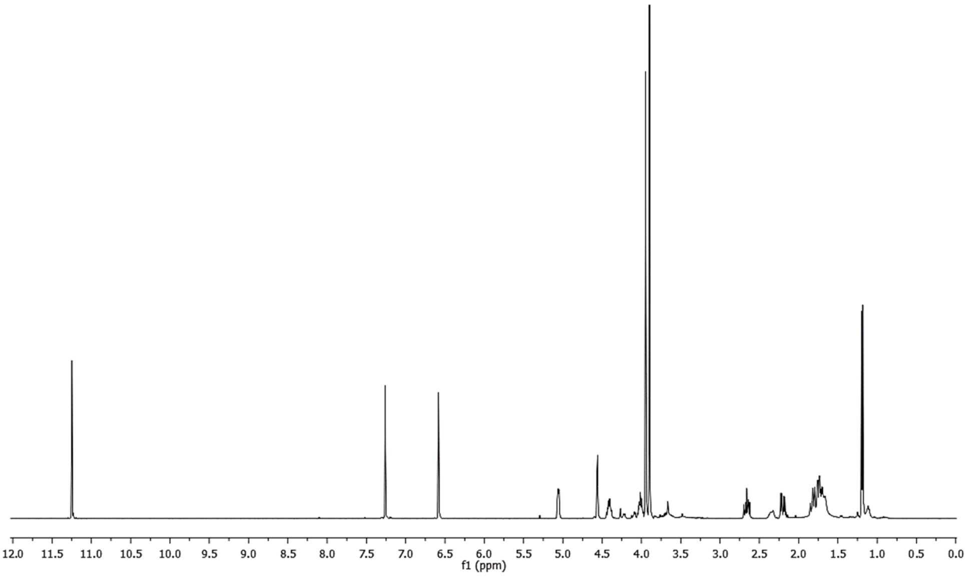

Fig. S7. ^{1}H NMR spectrum (400 MHz, $CDCl_3$) of setosphamarin B (**2**)

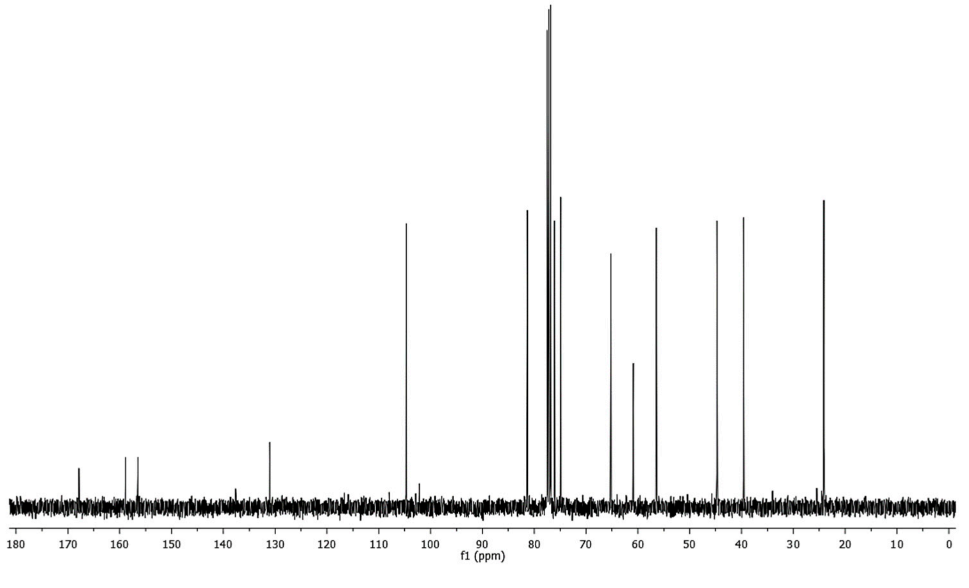

Fig. S8. ^{13}C NMR spectrum (100 MHz, $CDCl_3$) of setosphamarin B (**2**)

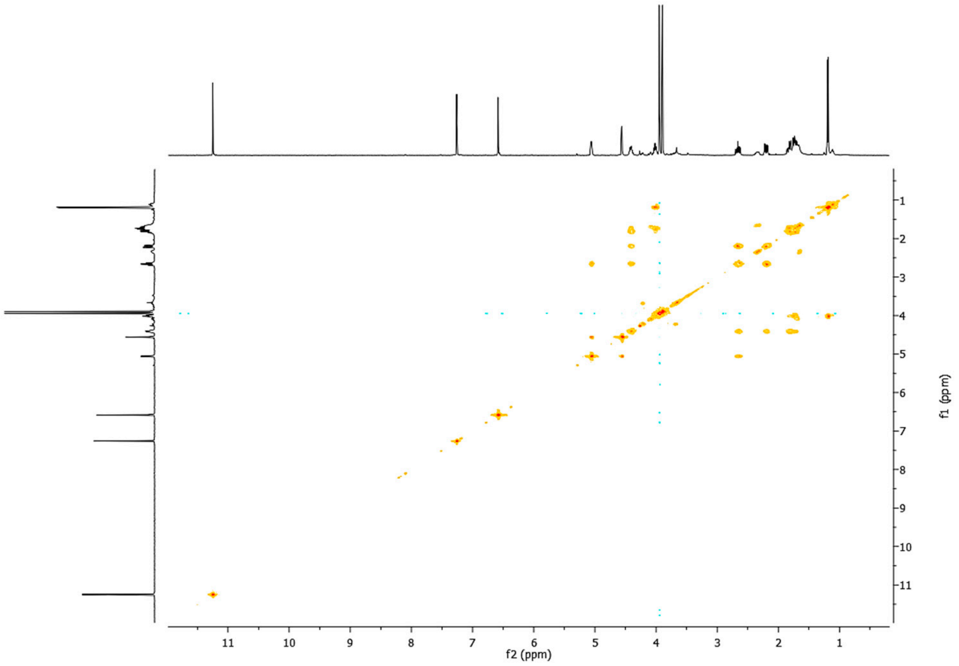

Fig. S9. COSY spectrum of setosphamarin B (**2**)

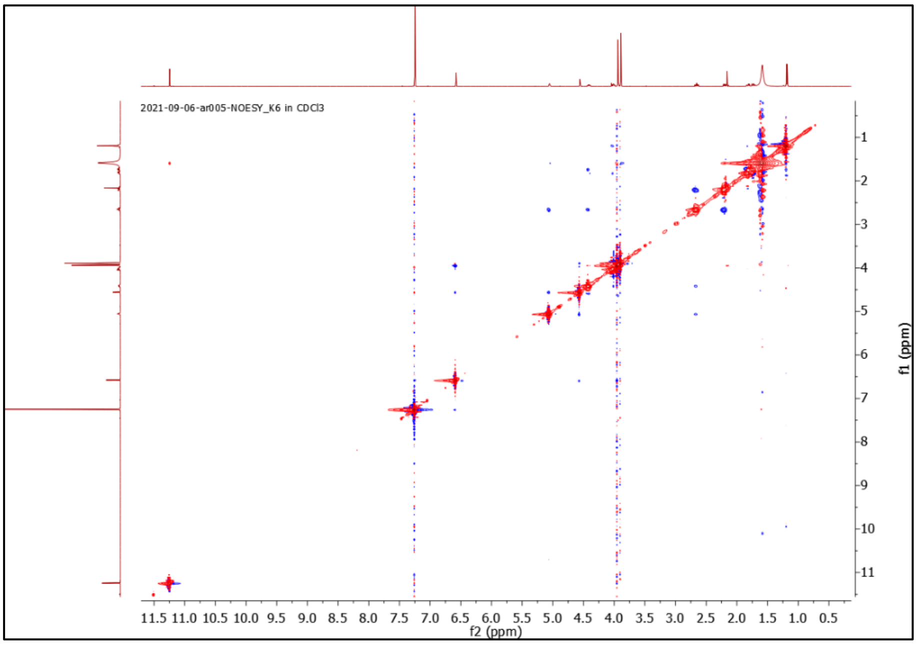

Fig. S10. NOESY spectrum of setosphamarin B (**2**)

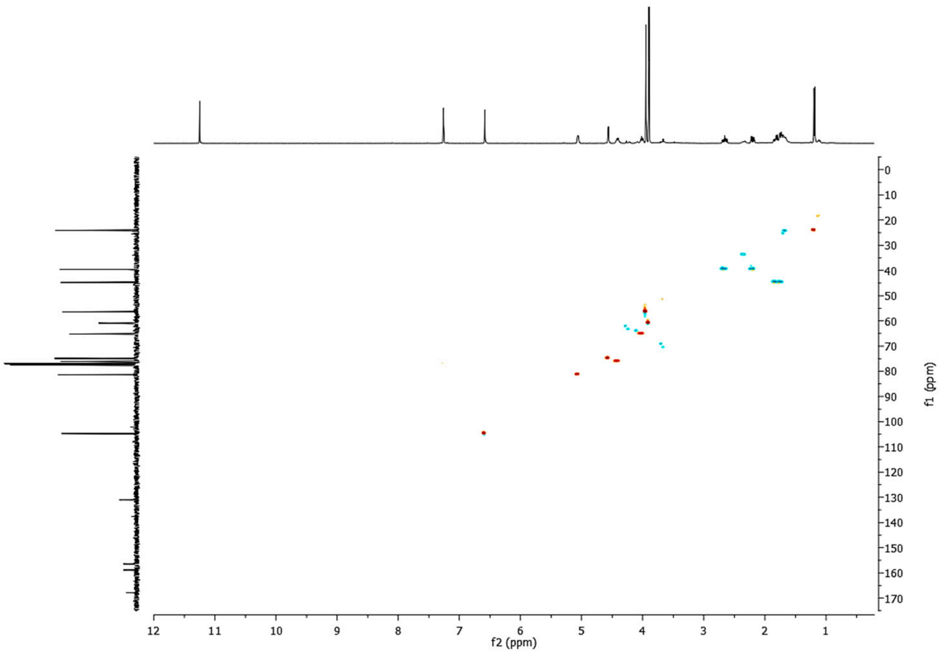

Fig. S11. HSQC spectrum of setosphamarin B (**2**)

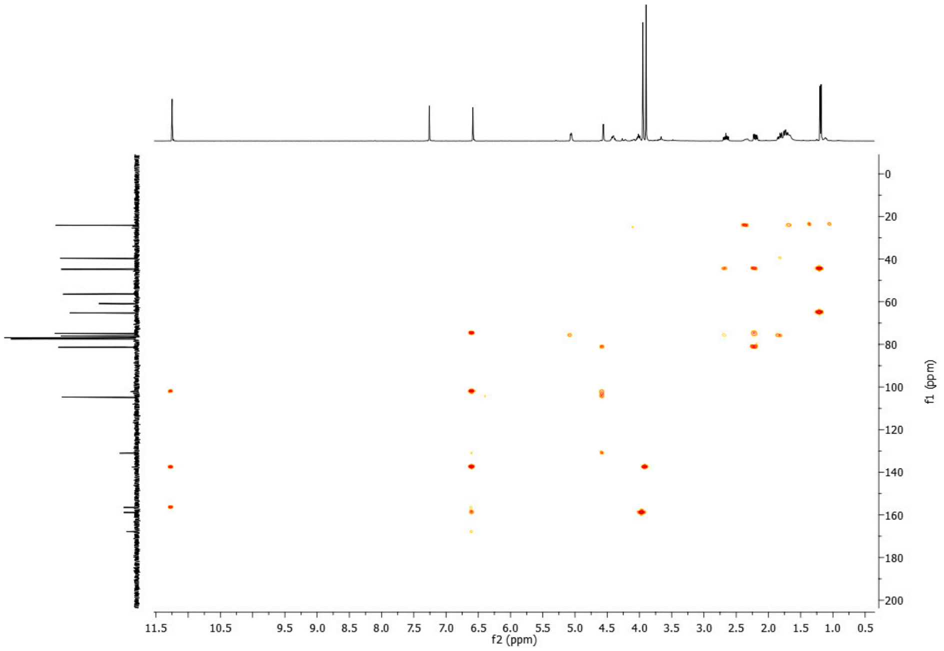

Fig. S12. HMBC spectrum of setosphamarin B (**2**)

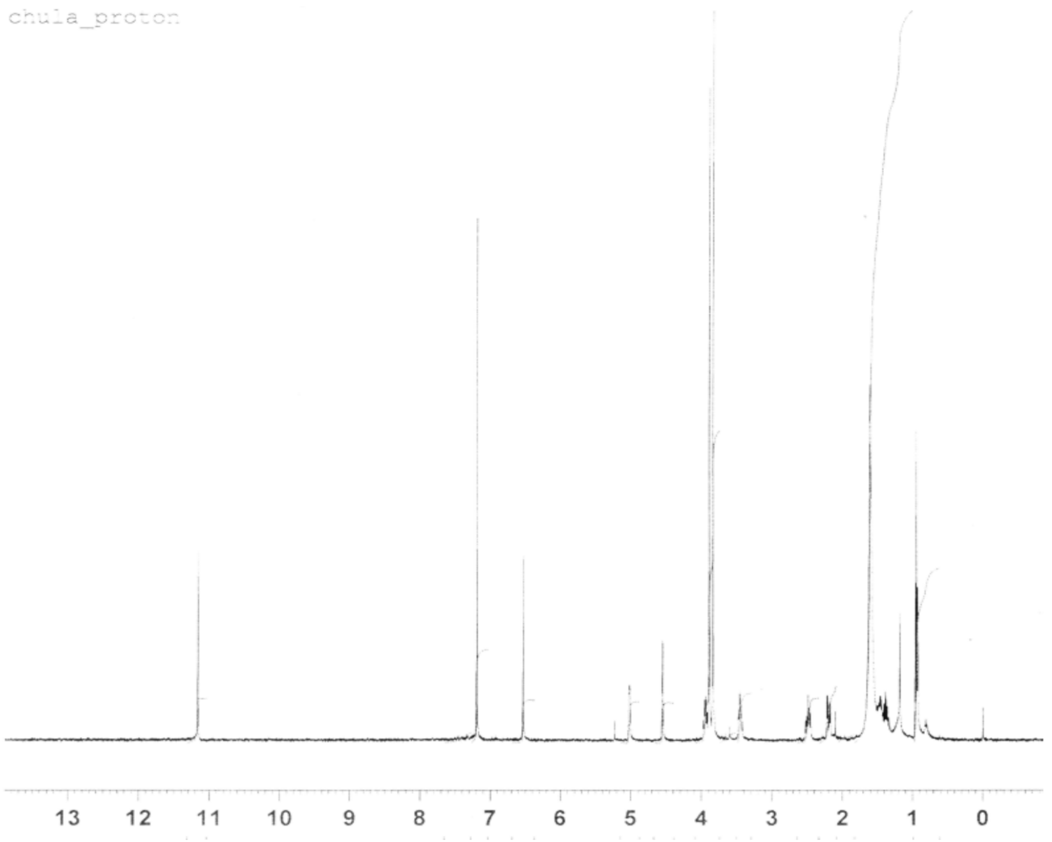

Fig. S13. ^{1}H NMR spectrum (400 MHz, $CDCl_3$) of setosphamarin C (**3**)

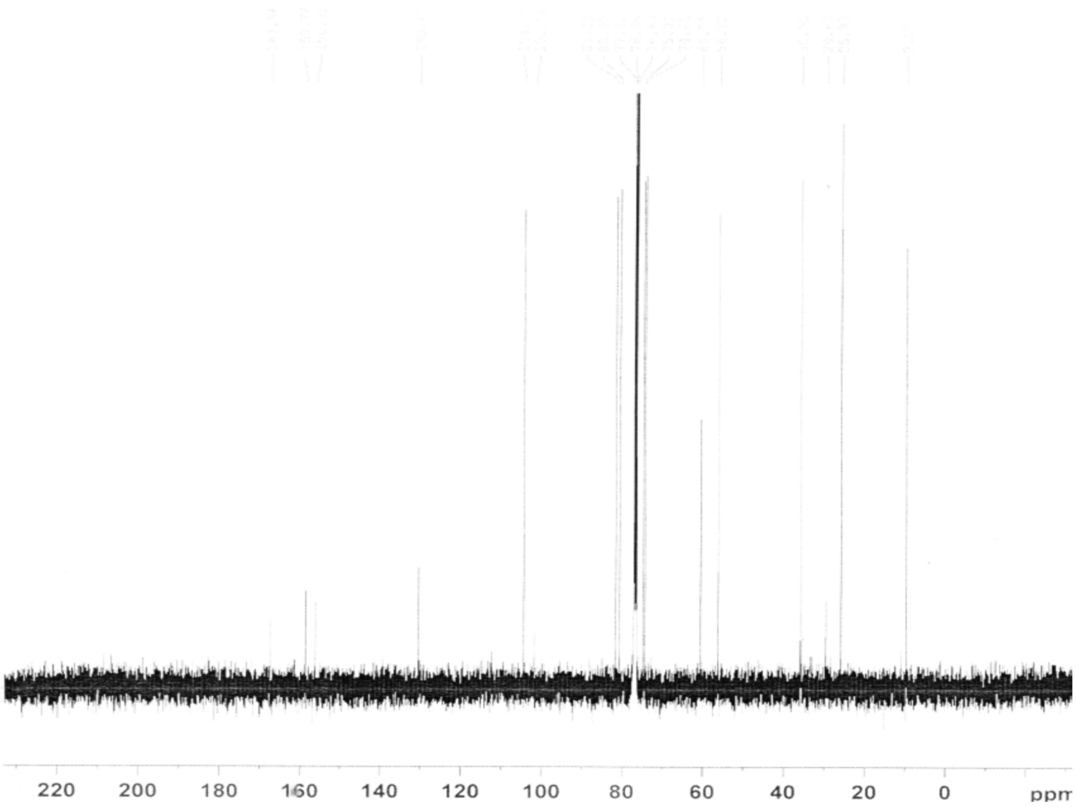

Fig. S14. ^{13}C NMR spectrum (100 MHz, $CDCl_3$) of setosphamarin C (**3**)

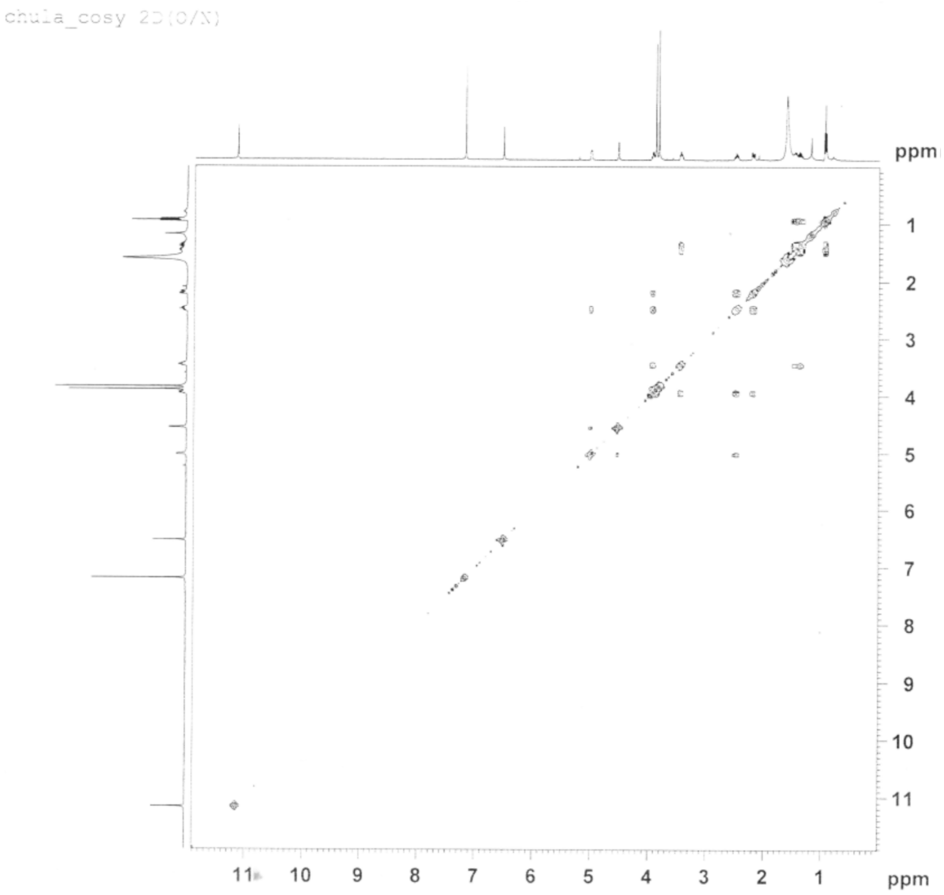

Fig. S15. COSY spectrum of setosphamarin C (**3**)

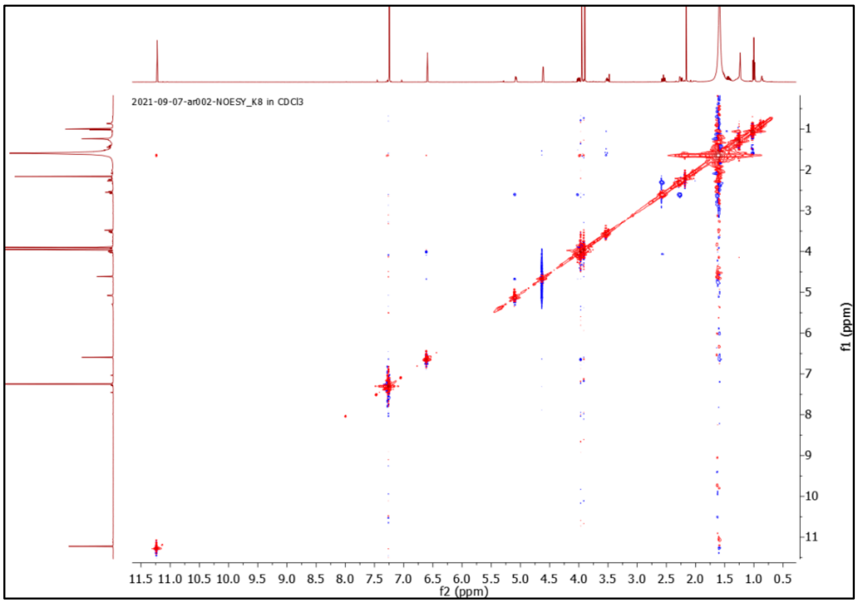

Fig. S16. NOESY spectrum of setosphamarin C (**3**)

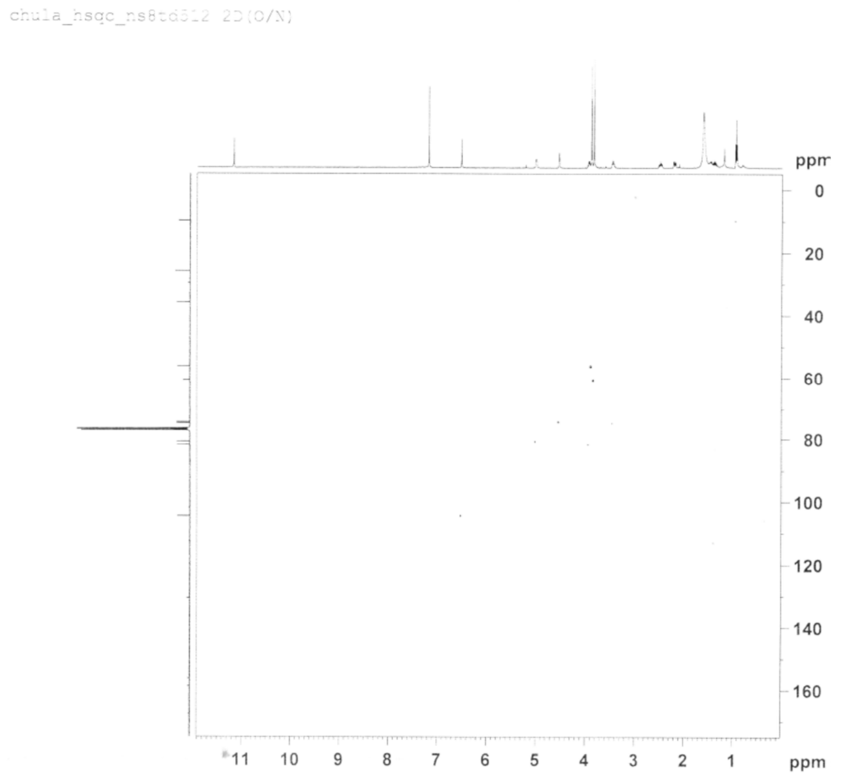

Fig. S17. HSQC spectrum of setosphamarin C (**3**)

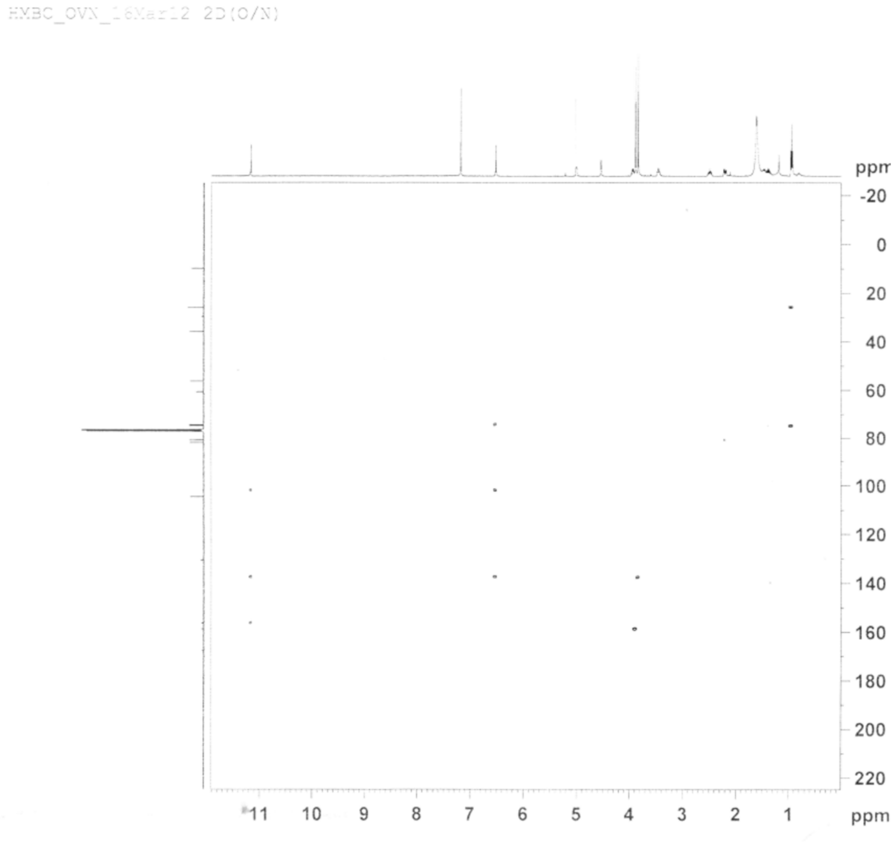

Fig. S18. HMBC spectrum of setosphamarin C (**3**)

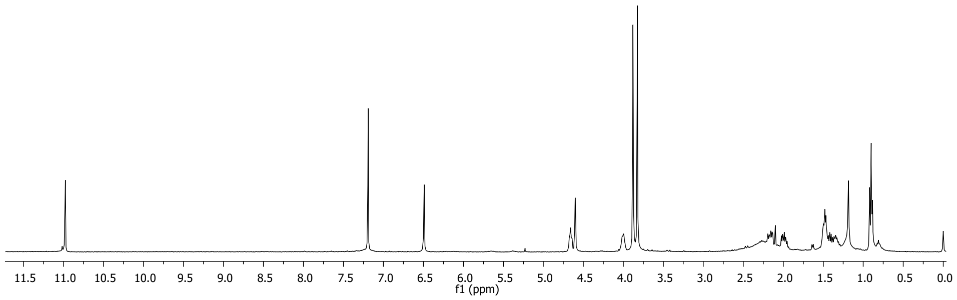

Fig. S19. ^{1}H NMR spectrum (400 MHz, $CDCl_3$) of setosphamarin D (**4**)

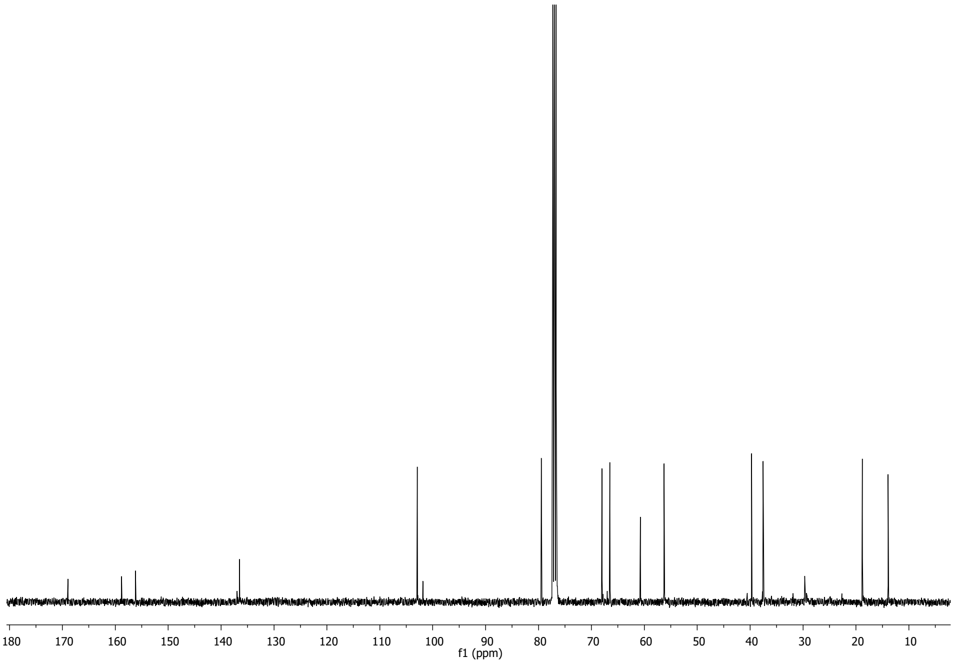

Fig. S20. ^{13}C NMR spectrum (100 MHz, $CDCl_3$) of setosphamarin D (**4**)

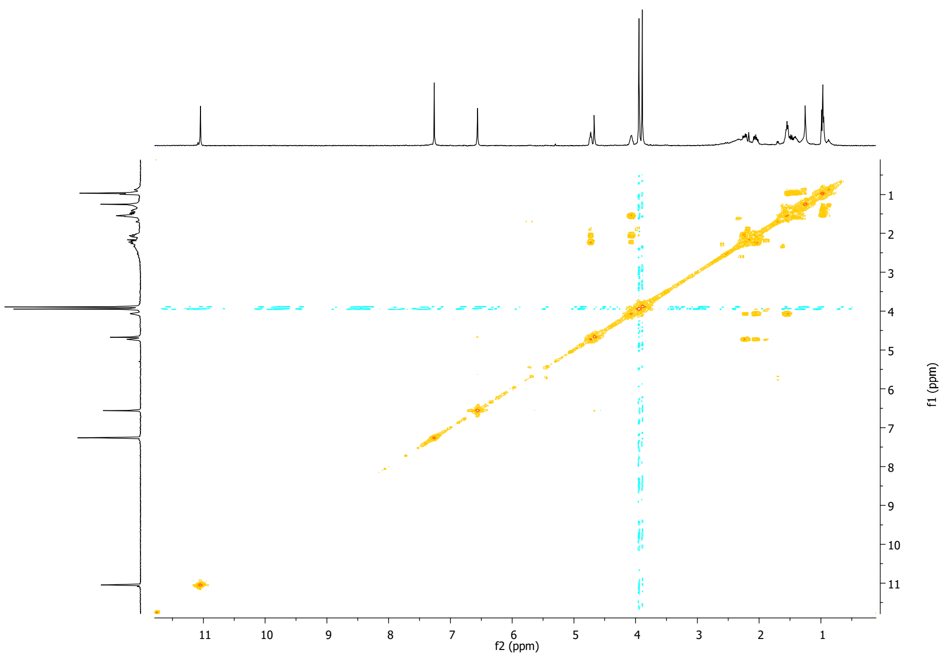

Fig. S21. COSY spectrum of setosphamarin D (**4**)

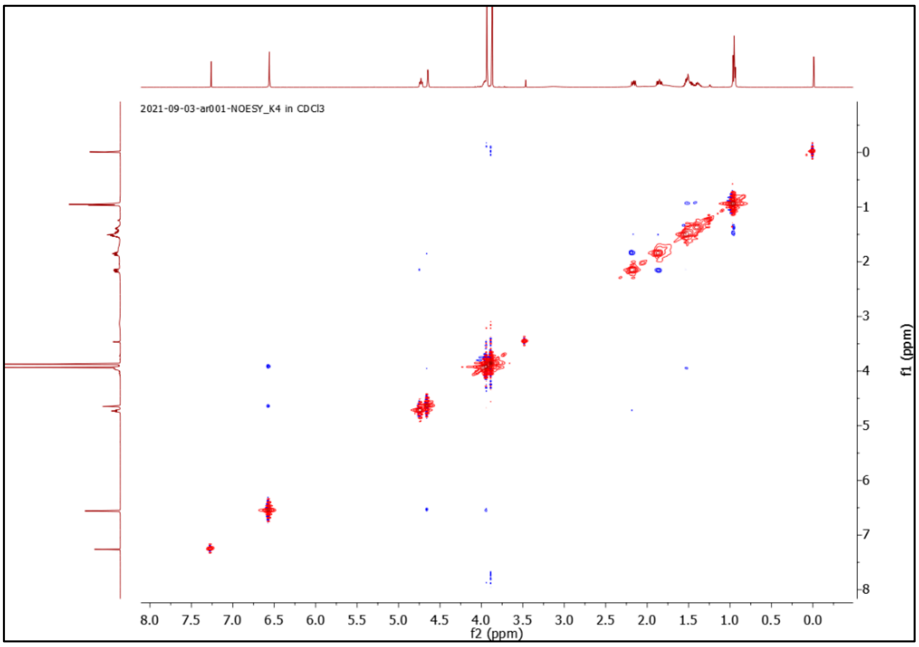

Fig. S22. NOESY spectrum of setosphamarin D (**4**)

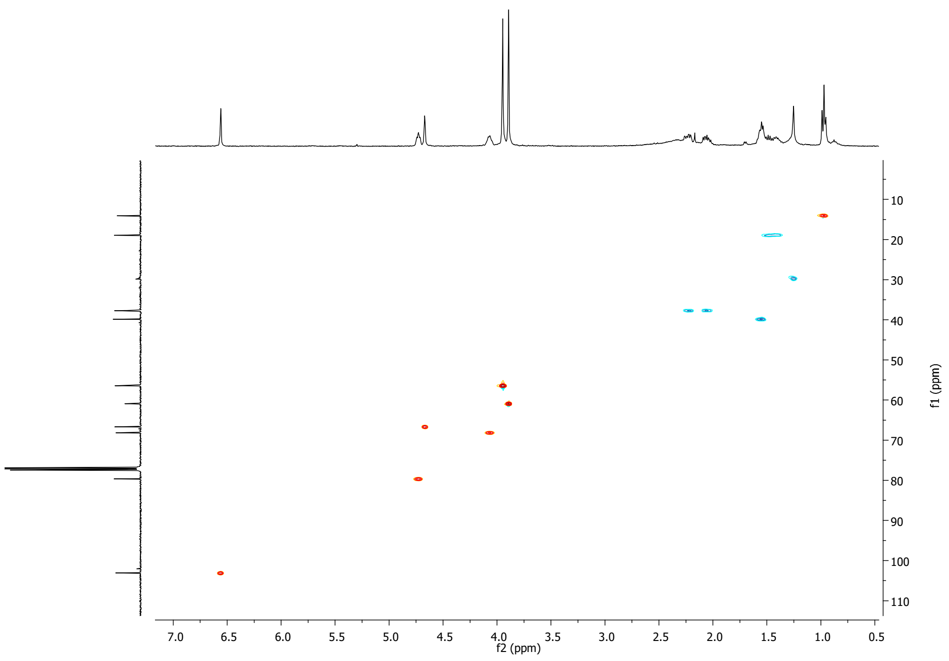

Fig. S23. HSQC spectrum of setosphamarin D (**4**)

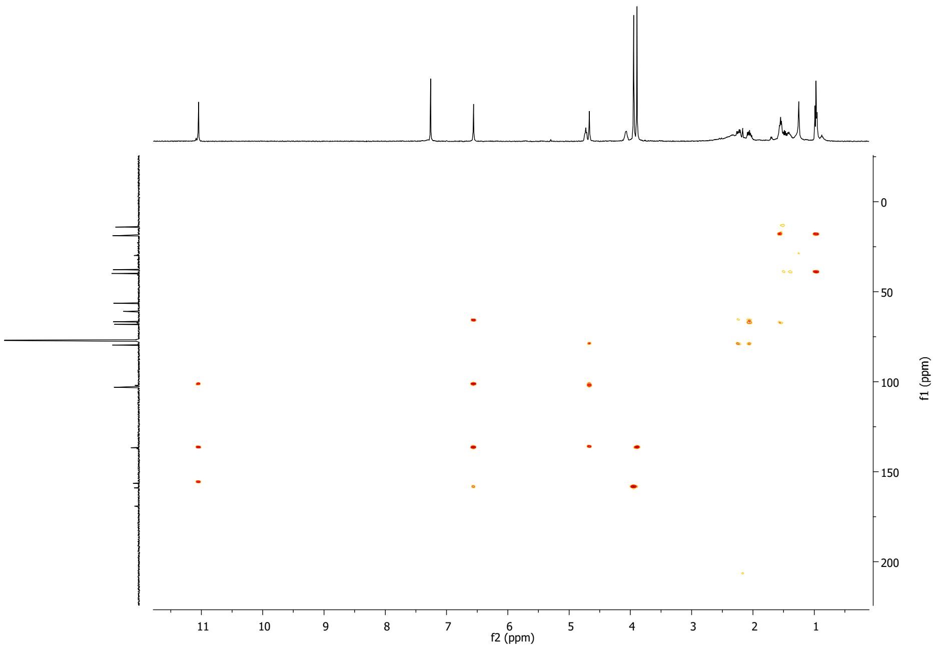

Fig. S24. HMBC spectrum of setosphamarin D (**4**)

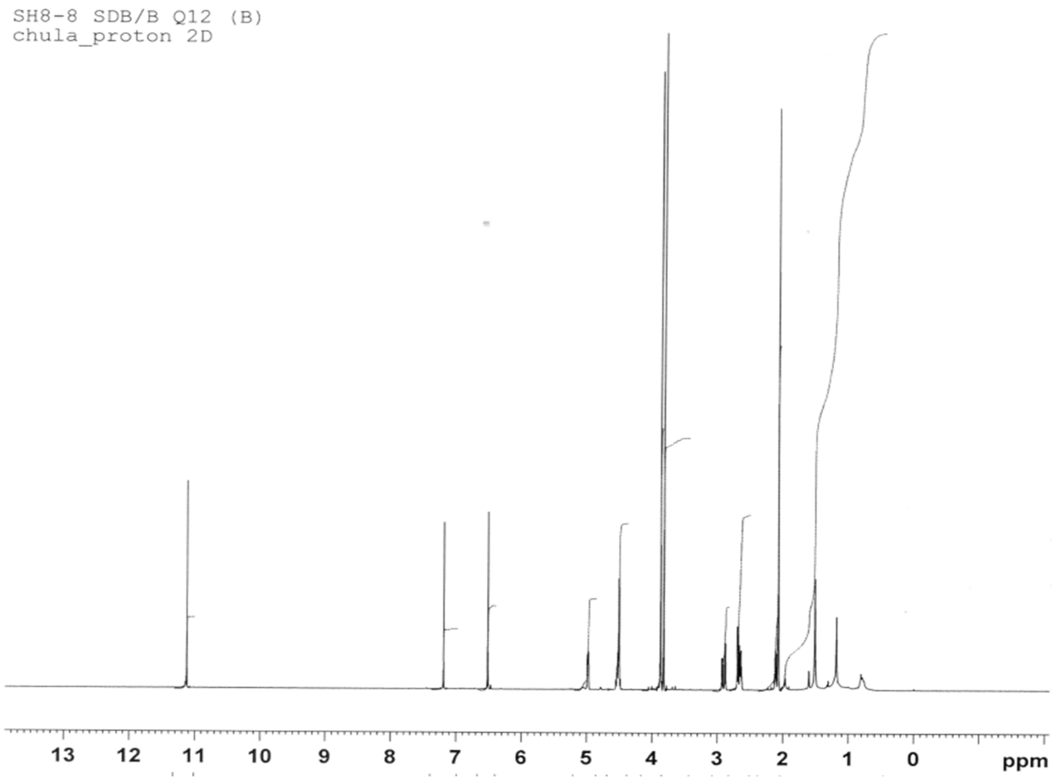

Fig. S25. ^{1}H NMR spectrum (400 MHz, $CDCl_3$) of setosphamarin E (**5**)

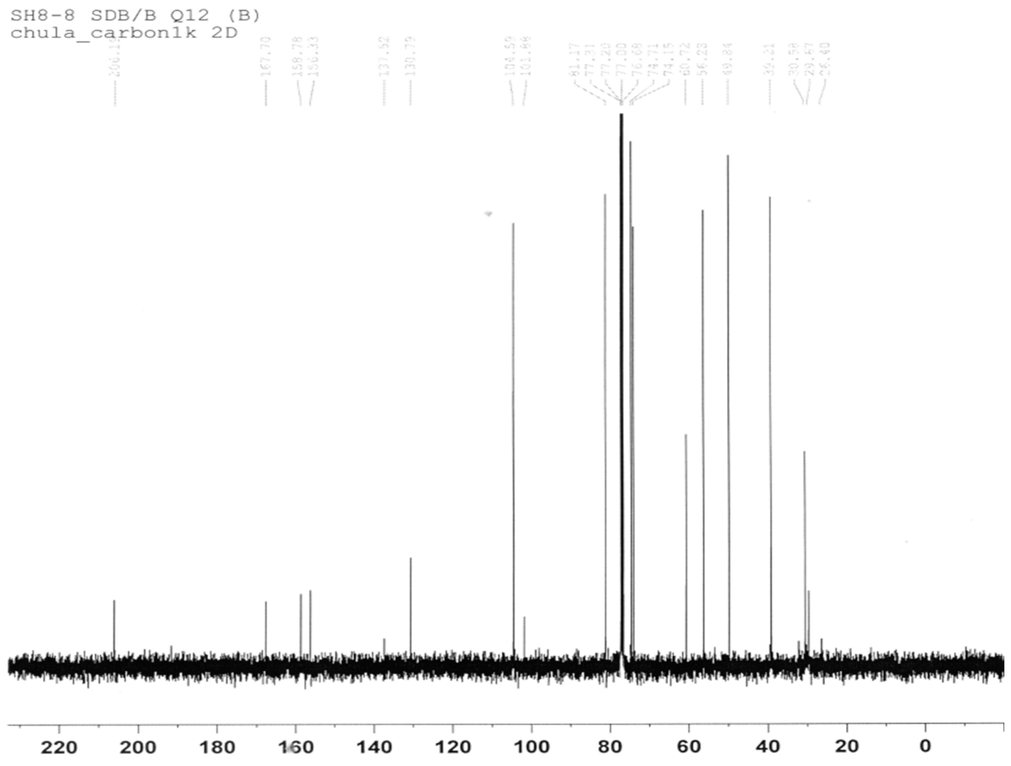

Fig. S26. ^{13}C NMR spectrum (100 MHz, $CDCl_3$) of setosphamarin E (**5**)

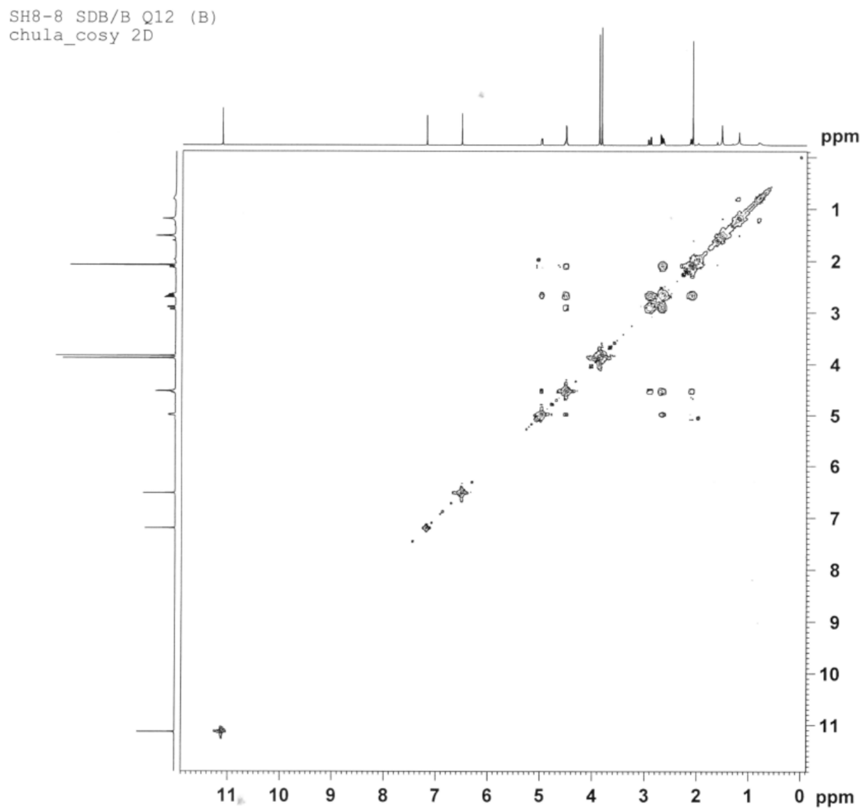

Fig. S27. COSY spectrum of setosphamarin E (**5**)

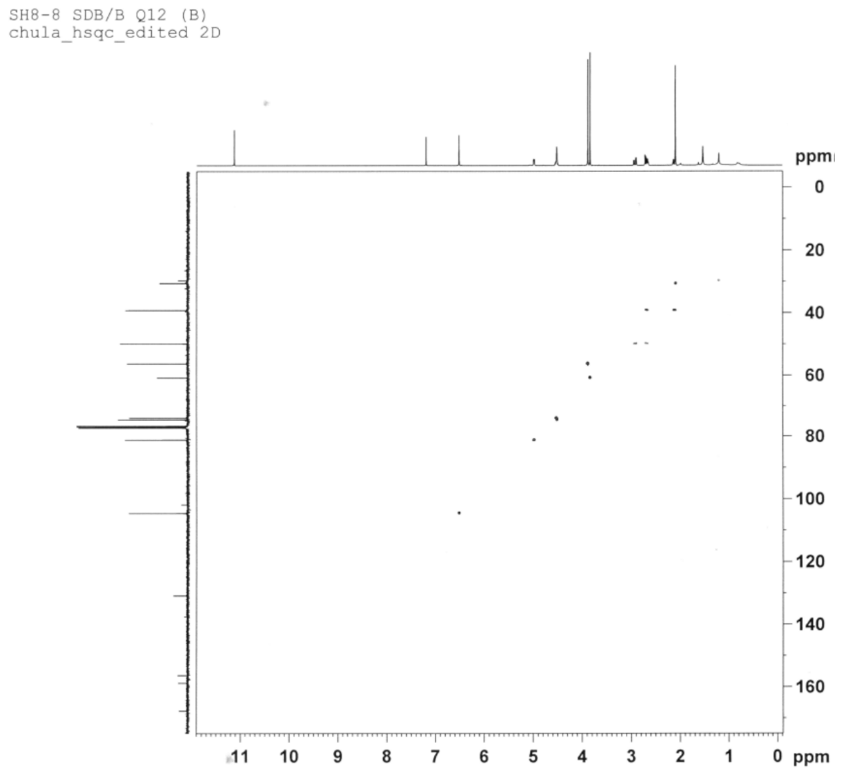

Fig. S28. HSQC spectrum of setosphamarin E (**5**)

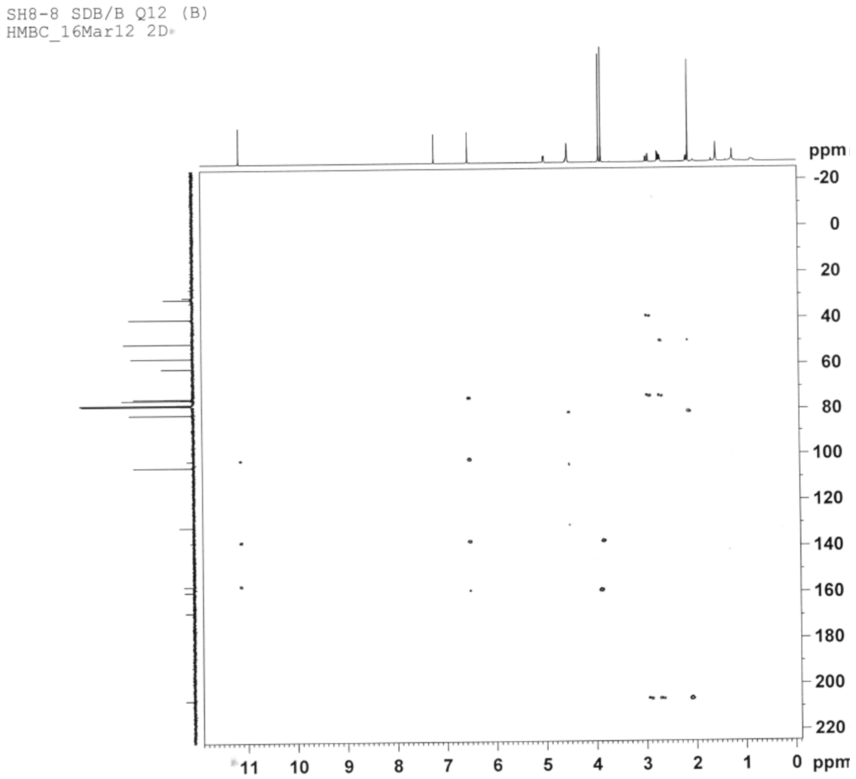

Fig. S29. HMBC spectrum of setosphamarin E (**5**)
